# Assessment of Bone Mineral Density in Pediatric Patients With Acute Leukemia

**DOI:** 10.7759/cureus.82685

**Published:** 2025-04-21

**Authors:** Steve Thomas, Manoranjan Mahapatra, Tulika Seth, Jyothsna Viveka

**Affiliations:** 1 Hematology, Sri Ramachandra Institute of Higher Education and Research, Chennai, IND; 2 Hematology, All India Institute of Medical Sciences, New Delhi, New Delhi, IND; 3 Endocrinology and Diabetes, All India Institute of Medical Sciences, New Delhi, New Delhi, IND

**Keywords:** acute lymphoblastic leukemia, bone mineral density, dual-energy x-ray absorptiometry, osteopenia, osteoporosis, pediatric patients

## Abstract

Background

Acute lymphoblastic leukemia (ALL) is the most prevalent highly curable childhood cancer, with long-term survival rates exceeding those of most other cancers. However, concerns persist regarding the adverse effects of treatment, particularly on bone health. Children with ALL often experience complications such as impaired growth, bone loss, and endocrine irregularities. This study aims to assess bone mineral density (BMD) in pediatric ALL patients undergoing chemotherapy, shedding light on the overlooked issue of osteoporosis.

Methodology

A prospective, observational study was conducted from February 2018 to August 2019. Children and adolescents (aged 2-20 years) with ALL or acute myelogenous leukemia undergoing induction chemotherapy were included. Baseline characteristics, nutritional parameters, and biochemical data were collected. BMD was assessed using dual-energy X-ray absorptiometry (DEXA).

Results

Among 64 patients, the majority were males (67.18%), and 39.06% were aged 16-20 years. Low serum albumin levels (20.31%) and deficiencies in folate (25.00%), vitamin B12 (28.13%), and vitamin D (95.31%) were observed. DEXA scans revealed that 46.43% had osteopenia, 28.57% had osteoporosis, and 25% had normal BMD. Serum calcium and vitamin D levels fluctuated but were not statistically significant. Duration from diagnosis to treatment did not differ significantly between patients with poor prognosis and those who achieved remission (p = 0.222).

Conclusions

This study underscores the need to monitor bone health in pediatric ALL patients, with a focus on early intervention and specialized care to manage osteopenia and osteoporosis. Comprehensive nutritional and metabolic assessments are essential for their well-being during leukemia treatment.

## Introduction

Acute lymphoblastic leukemia (ALL), a malignancy of T and B lymphoblast cells, is generally characterized by uncontrolled and abnormal proliferation of immature lymphocytes, which act as progenitors, ultimately, leading to bone marrow replacement and lymphoid organ replacement [1]. It is the most common curable childhood cancer, with long-term survival rates exceeding 90% [2]. Environmental factors include exposure to ionizing radiation, chemotherapy, radiotherapy, and/or benzene. The genetic factors, such as somatic, polymorphic variants of *ARD5B*, *IKZF1* (the gene encoding Ikaros), and *CDKN2A*, are associated with increased risk of ALL [1]. While survival rates for pediatric patients with ALL have significantly improved, there is still a significant concern about the adverse effects of treatment. These patients often suffer from enduring side effects that have a lasting impact on their quality of life, with the most prevalent skeletal complications being bone loss and impaired longitudinal growth. The standard treatment for ALL is risk-directed therapy, with two to three years of chemotherapy [3]. Children undergoing treatment for ALL may experience endocrine complications such as impaired growth, elevated blood sugar levels, thyroid irregularities, adrenal insufficiency, gonadal dysfunction, inappropriate secretion of antidiuretic hormone, and reduced bone mineral density (BMD) [4,5]. Multiple studies have indicated that pediatric ALL survivors are at a heightened risk of bone toxicity when compared to survivors of other cancer types. This increased risk may stem from leukemia infiltration affecting vitamin D metabolism, coupled with exposure to high doses of corticosteroids and methotrexate [6]. Dietary problems and decreased physical activity are additional factors that influence bone health [7].

Osteopenia and osteoporosis are often overlooked issues that can arise from both the disease and the use of chemotherapy agents. Osteoporosis leads to a diminished quality of life, heightened disability-adjusted years, and significant economic strain [8]. Therefore, the early detection of osteoporosis is necessary [9]. The gold standard for diagnosing osteoporosis involves assessing BMD through dual-energy X-ray absorptiometry (DEXA) [10]. There are only a few Indian studies reported the assessment of BMD through DEXA in the pediatric and adolescent ALL population [11-13]. Therefore, the present study aimed to assess BMD with DEXA in children with ALL.

## Materials and methods

Study design

This prospective, observational study was conducted at the hematology wards in All India Institute of Medical Sciences (AIIMS), New Delhi, over 18 months between February 2018 and August 2019. The study was approved by the Institutional Ethics Committee (approval number: IECPG-77/28.02.2018), and was performed in accordance with the Declaration of Helsinki and the International Conference on Harmonization guidelines. Written informed consent was obtained from all participants before enrollment in this study.

Study participants

The study included children and adolescents (aged 2-20 years) of either sex diagnosed with acute leukemia (ALL/acute myeloid leukemia (AML)) and undergoing induction chemotherapy in hematology wards. Those who had received partial treatment from elsewhere and had relapsed disease were excluded from the study.

Data collection

Baseline characteristics such as height, weight, and body mass index (BMI) were recorded along with nutritional parameters such as micronutrients (calcium, vitamin D, phosphorus) at admission, after two weeks, and after four weeks (at the end of induction).

BMI assessment

BMI was calculated using the following formula: weight (kg)/height^2^ (cm^2^). The following BMI percentile interpretation (World Health Organization (WHO)) [14] was used: percentile <5: underweight; percentile ≥5 and <85: healthy weight; percentile ≥85 and <95: risk for overweight; percentile ≥95: overweight.

BMD measurement

DEXA was performed using the Hologic Discovery QDR Series at admission. Bone mineral content (g), bone area (cm^2^), and BMD (bone mineral content divided by bone area, g/cm^2^) measurements were performed using DEXA (Osteocor 2, France) in the lumbar spine (L2-L4), femoral neck, and forearm. BMD measurements were compared with age, sex, and race-specific normative values provided by Osteocor 2, pediatric software, and expressed as Z-scores. To minimize the effect of bone size on BMD values, bone mineral apparent density (g/cm^3^) was calculated for both the lumbar spine and femoral neck by dividing bone mineral content by bone area to the power of 1.5 and 2, respectively [15].

In accordance with the criteria established by WHO, a Z-score equal to or greater than -1 was classified as normal bone density, while a Z-score between -1 and -2.5 signified osteopenia, indicating a reduction in BMD. A Z-score equal to or less than -2.5 indicated osteoporosis, indicating a significant decline in BMD. These defined thresholds were applied in this study [16].

Statistical analysis

Data analysis was performed using SPSS Statistics version 21.0 (IBM Corp., Armonk, NY, USA). Descriptive data were expressed as median with interquartile range for continuous data or as frequency (percentage) for categorical data. Categorical variables were compared using chi-square tests. Continuous variables were compared using the Mann-Whitney (two-group comparison) and Kruskal-Wallis (multiple-group comparison) non-parametric tests. P-values <0.05 were considered statistically significant.

## Results

The study population consisted of 64 patients diagnosed with ALL and a median age of 14.5 years. Among these patients, the largest proportion, 39.06%, fell within the 16-20-year age group, followed by 32.8% in the 11-15-year age group, 20.3% in the 1-5-year age group, and 7.81% in the 6-10-year age group. The majority of the patients (67.18%) were male, while 32.81% were female. BMI measurements revealed that 45.31% of patients were underweight, while 51.56% maintained a healthy weight, and 3.13% were overweight. Concerning clinical diagnoses, the majority (59.37%) had been diagnosed with B-cell ALL, followed by 20.31% with AML, 9.37% with T-cell ALL, 7.81% with acute promyelocytic leukemia, and 3.12% with B-cell mixed phenotype acute leukemia (Table 1).

**Table 1:** Demographic and baseline characteristics. Data presented as n (%). BMI: body mass index; AML: acute myelogenous leukemia; APML: acute promyelocytic leukemia; B12: cobalamin; B-ALL: B-cell acute lymphoblastic leukemia; B-MPAL: B-cell mixed phenotype acute leukemia; T-ALL: T-cell acute lymphoblastic leukemia; TIBC: total iron-binding capacity

Patient characteristics	Normal ranges	Number of patients (N = 64)
Age group (years)		
1–5	_	13 (20.3)
6–10		5 (7.81)
11–15		21 (32.8)
16–20		25 (39.06)
Sex		
Male	_	43 (67.18)
Female		21 (32.81)
BMI (kg/m^2^)		
Underweight	18.5–24.9	29 (45.31)
Healthy weight		33 (51.56)
Overweight		2 (3.13)
Clinical diagnosis		
B-ALL	_	38 (59.37)
AML		13 (20.31)
T-ALL		6 (9.37)
APML		5 (7.81)
B-MPAL		2 (3.12)
Hyperleukocytosis (/L)		
Yes	4,500–11,000	18 (28.12)
No		46 (71.87)
Serum albumin (g/dL)		
Low	3.4–5.4	13 (20.31)
Normal		51 (79.68)
Folate (ng/mL)		
Low	4.0–17.0	16 (25.00)
Normal		48 (75.00)
Vitamin B12 (pg/mL)		
Low	174–878	18 (28.13)
Normal		46 (71.88)
Serum iron (µg/dL)		
Low	60–180	4 (6.25)
Normal		60 (93.75)
Serum ferritin (ng/mL)		
Normal	Males = 23–336; females = 11–306	10 (15.63)
High		54 (84.38)
TIBC (µg/L)		
Low	155–355	9 (14.06)
Normal		55 (85.94)
Vitamin D (ng/mL)		
Low	30–100	61 (95.31)
Normal		3 (4.69)
Serum calcium (mg/dL)		
Low	8.5–10.2	36 (56.25)
Normal		28 (43.75)
Phosphorus (mg/dL)		
Low	2.5–4.5	3 (4.69)
Normal		61 (95.31)

Hyperleukocytosis was present in 28.12% of patients, while 71.87% of patients did not have hyperleukocytosis. Low serum albumin levels were observed in 20.31% of patients, along with low folate levels (25.00%) and low vitamin B12 levels (28.13%). Low serum iron was found in a small proportion of patients (6.25%), along with elevated serum ferritin (84.38%) and total iron binding capacity (14.06%). A majority of patients had low vitamin D levels (95.31%), followed by low serum calcium levels (56.25%), and a small proportion exhibited low phosphorus levels (4.69%).

During the study, changes in serum calcium levels were observed over a four-week period. At week zero, the mean serum calcium level was 8.36 mg/dL, which decreased to 8.19 mg/dL at week two but increased to 8.43 mg/dL at week four. However, these changes were not statistically significant (p > 0.05). The mean vitamin D level was 12.2 ng/mL at week zero, which decreased to 10.6 ng/mL at week two and slightly increased to 10.9 ng/mL at week four (p > 0.05) (Table 2).

**Table 2:** Trends of serum calcium and vitamin D over the study period. Data presented as mean (SD), unless otherwise specified. The Kruskal-Wallis non-parametric test was used to perform multiple group comparison. ^a^: P-value for comparison of parameters from baseline to week two. ^b^: P-value for comparison of parameters from baseline to week four.

Parameters	Normal ranges	Week 0	Week 2	Week 4	P-value
Serum calcium (mg/dL)	8.5–10.2	8.36 (0.67)	8.19 (0.81)	8.43 (0.96)	0.175^a^
					0.252^b^
Vitamin D (ng/mL), median (range)	30–100	12.2 (3–45.3)	10.6 (4–53)	10.9 (4–55.2)	0.236^a^
					0.883^b^

Of the 64 patients in the study, only 56 underwent a DEXA scan. Dexa Z-score measurements indicated 46.43% of patients exhibited osteopenia, 28.57% were diagnosed with osteoporosis, and 25% had normal BMD. For most patients, the site of bone density assessment was the lumbar spine (53.57%), 25% at the hip, and 21.43% of patients at the forearm (Table 3).

**Table 3:** Outcomes of DEXA scan. Data presented as n (%), unless otherwise specified. BMD: bone mineral density; DEXA: dual-energy X-ray absorptiometry

Parameters	Number of patients (N = 56)
DEXA Z-score	
Normal BMD (g/cm^2^)	14 (25)
Osteopenia	26 (46.43)
Osteoporosis	16 (28.57)
Site	
Forearm	12 (21.43)
Hip	14 (25.00)
Lumbar spine	30 (53.57%)

Following induction chemotherapy, 75% of the patients successfully achieved remission, while the remaining 25% had persistent disease and mortality. The study found that among the 48 patients without a poor prognosis, the median duration from diagnosis to treatment was 20 days, while for those with a poor prognosis (n = 16), it was 24.5 days, with no statistically significant difference between the two groups (p = 0.222).

Table 4 represents BMD scores according to BMI at weeks zero, two, and four. At week zero, there was no significant difference in BMD Z-scores among BMI categories (p = 0.557). However, by week two and week four, there was a significant association between higher BMI categories and BMD Z-scores (p < 0.001).

**Table 4:** BMD scores according to BMI at weeks zero, two, and four. Data are presented as n (%). The Kruskal-Wallis non-parametric test was used to perform multiple group comparisons. BMD: bone mineral density; BMI: body mass index

BMI	BMD Z-score			P-value
	Normal BMD	Osteopenia	Osteoporosis	
At week 0	n = 16	n = 28	n = 12	0.557
Underweight	8 (50.0)	11 (39.3)	7 (58.3)	
Healthy weight	8 (50.0)	16 (57.1)	4 (33.3)	
Overweight	0	1 (3.6)	1 (8.3)	
At week 2	n = 21	n = 16	n = 27	<0.001
Underweight	0	11 (68.8)	27 (100)	
Healthy weight	19 (90.5)	5 (31.3)	0	
Overweight	2 (9.5)	0	0	
At week 4	n = 17	n = 18	n = 23	<0.001
Underweight	0	12 (66.7)	23 (100)	
Healthy weight	14 (82.4)	6 (33.3)	0	
Overweight	3 (7.6)	0	0	

## Discussion

Individuals who survived cancer during their early years face a risk of developing long-term health complications such as bone pain, bone deformity, and fractures [17]. Osteopenia and osteoporosis are frequently disregarded concerns that can result from both the underlying disease and the administration of chemotherapy agents. Osteoporosis leads to a diminished quality of life, heightened disability-adjusted years, and economic burden [8]. Therefore, the early detection of osteoporosis is necessary [9]. Even though DEXA is the gold standard for the diagnosis of osteoporosis, it is still underutilized in developing countries such as India [10]. Therefore, this study aimed to investigate BMD using DEXA in children with ALL and the key findings were (i) most patients belonged to the age group of 16-20 years and male predominance; (ii) the majority were diagnosed with B-cell ALL, followed by AML and T-cell ALL; (iii) a small proportion of patients had hyperleukocytosis; (iv) a majority of patients had deficiencies in vitamin D, calcium, and phosphorus; (v) DEXA Z-score revealed that the majority were diagnosed with osteoporosis, followed by osteopenia; (vi) the most prevalent site for BMD assessment was the lumbar spine, followed by the hip and forearm; (vii) no significant difference was observed between median duration from diagnosis to treatment; and (viii) at weeks two and four, BMI significantly varied according to the BMD Z-scores.

Within this patient cohort, the majority (39.06%) belonged to the 16-20-year age group, while 32.8% were in the 11-15-year age group. Additionally, 20.3% fell within the age group of 1-5 years, and 7.81% were in the 6-10-year age group. The mean age of the patients with ALL was 7.06 years. In another study, it was found that the male to female ratio in ALL patients was 2:1 [18], which was similar to our study with 67.18% males and 32.81% females.

In this study, the most prevalent type of ALL was B-cell ALL. The prevalence of B-cell ALL in children was approximately 30% of all the cancers in the United States [3]. While in India, the prevalence was pre-B ALL (88.6%), B-cell ALL in 1.7%, and T-cell ALL in 9.7% of patients [19]. The common ALL phenotype comprised most cases, although the incidence of T-cell ALL was notably high at 27.9% [20]. Similarly, other studies have reported that the most prevalent type of ALL, known as B-cell ALL in the age range of 2 to 5 years [21,22].

The initial leukocyte count in patients with ALL significantly influences the achievement of complete remission during induction and the subsequent event-free survival [23]. Hyperleukocytosis resulting from ALL, characterized by elevated white blood cell counts, leads to increased morbidity and mortality due to blood thickening (hyperviscosity) caused by excessive leukocyte levels. These patients face up to a 20% mortality risk during remission induction therapy [23]. In this study, 28.12% of patients had hyperleukocytosis, which was comparatively higher compared to the results of another study reporting 8.9% of patients [20]. A comparable pattern was observed in the research conducted by Gustaitė et al. [24], where elevated white blood cell counts during the initial manifestation of acute leukemia were associated with an increased risk of early mortality, often attributed to leukostasis. Similarly, Oliviera et al. [25] studied hyperleukocytosis and revealed a markedly reduced overall survival (p < 0.0001) and a higher occurrence of premature deaths (p = 0.0008). Vitamin D plays a crucial role in regulating calcium and phosphorus levels to support bone health during childhood and adolescence [26]. Low vitamin D status can have an impact on adverse medical outcomes in childhood cancer, potentially increasing the risk of osteoporosis and cardiovascular disease in survivors of childhood malignancies [27]. In this study, vitamin D deficiency was found in 95.31% of patients, which was consistent with another study of ALL patients reporting vitamin D deficiency in 84.95% of patients [28]. A study by Bhattacharya et al. [28] reported significantly low vitamin D levels in children with ALL who experienced complications and mortality (p < 0.05 for both). In this study, fluctuations were seen in the vitamin D levels during the study period, with no statistical significance. Conversely, another study reported a significant drop in vitamin D levels when comparing pre- and post-induction (p < 0.001) in patients with ALL [28]. Contrary to our study findings, a previous study reported fewer patients in the age group of 9-18 years showed abnormalities in vitamin D (31.1%), calcium (32.3%), and phosphorus (23.2%) in ALL survivors [29]. This underscores a significant inadequacy in nutrient intake in this population, emphasizing the importance of targeted dietary interventions to support their long-term health, particularly concerning bone health. Alternative explanations for reduced BMD in these patients encompass disease pathology, growth hormone or sex hormone deficiencies, intensive chemotherapy, low calcium and vitamin D intake, and decreased physical activity [30]. In a previous study involving a sizable and varied cohort of childhood cancer survivors, a significant prevalence of 25-OH vitamin D insufficiency, reaching 29%, was reported [27].

Early detection of bone abnormalities is made easy with DEXA scan, yet in developing countries, it remains underutilized for diagnosis. Previous studies have reported the prevalence rates of low BMD in cancer survivors spanning from 8% to 51% [2,31-33]. In this study, we reported majority of patients (46.43%) had osteopenia and 28.57% had osteoporosis. Another study reported that 38% of patients had a normal BMD according to Z-scores, 50% had low bone density, and 12% were diagnosed with osteoporosis [34]. In contrast to this study, Ghassemi et al. reported that all 25 patients with ALL had abnormal BMD at the lumbar spine (L2-L4), with three (6%) patients having osteopenia, and 22 (44%) patients exhibiting osteoporosis [16].

In the present study, the site of BMD assessment was the lumbar spine in 53.57%, the hip in 25%, and the forearm in 21.43% of patients. A previous study indicated that for children aged 0-5 years, feasible measurement sites include the lumbar spine, while for those aged three years and older, whole-body measurements are viable [4]. The logistic regression analysis of a previously conducted study revealed that an elevated lumbar spine BMD Z-score yielded an odds ratio of 1.8 (95% confidence interval = 1.10 to 2.9, p < 0.001), indicating a significant association with an increased risk of fractures [35]. Therefore, the early detection of low BMD is necessary for selecting appropriate and timely therapeutic intervention.

The induction phase lasts approximately four weeks and aims to achieve remission, traditionally defined as having fewer than 5% bone marrow blasts based on morphology [36]. In this study, after induction chemotherapy, 75% of the patients successfully achieved remission. The median duration from diagnosis to treatment was higher in those with poor prognosis compared to those with complete remission; however, this was not statistically significant. Likewise, response to treatment was achieved in four weeks in 66.6% of patients in a study conducted by Khalid et al. [37].

In this study, at weeks two and four, a significant variation was observed in patients regarding BMD Z-scores (p < 0001). Likewise, a study conducted among cancer survivors reported that a reduction in BMI was a risk factor for decreased BMD [38].

Study limitations

This study has a few limitations, including a small sample size, potential bias due to excluding patients with prior treatment, a short-term focus that may not capture long-term changes in bone health, and the absence of a control group, making it challenging to establish causal links.

## Conclusions

This study highlights the significance of monitoring bone health in pediatric patients diagnosed with acute leukemia. The prevalence of osteopenia and osteoporosis, as revealed by DEXA scans, underscores the need for early intervention and specialized care to mitigate the long-term skeletal complications that these young patients may face. It also emphasizes the necessity of comprehensive nutritional assessments, given the observed deficiencies in key nutrients (vitamin D, calcium, vitamin B12, folate). Additionally, this study underscores the importance of considering bone health in the comprehensive care of pediatric ALL patients to enhance their overall well-being and quality of life.

## References

[1] Puckett Y, Chan O (2025). Acute Lymphocytic Leukemia. https://www.ncbi.nlm.nih.gov/books/NBK459149/.

[2] Al-Mahayri ZN, AlAhmad MM, Ali BR (2021). Long-term effects of pediatric acute lymphoblastic leukemia chemotherapy: can recent findings inform old strategies?. Front Oncol.

[3] Huang FL, Liao EC, Li CL, Yen CY, Yu SJ (2020). Pathogenesis of pediatric B-cell acute lymphoblastic leukemia: molecular pathways and disease treatments. Oncol Lett.

[4] Ahn MB, Suh BK (2020). Bone morbidity in pediatric acute lymphoblastic leukemia. Ann Pediatr Endocrinol Metab.

[5] Marcucci G, Beltrami G, Tamburini A (2019). Bone health in childhood cancer: review of the literature and recommendations for the management of bone health in childhood cancer survivors. Ann Oncol.

[6] Wasilewski-Masker K, Kaste SC, Hudson MM, Esiashvili N, Mattano LA, Meacham LR (2008). Bone mineral density deficits in survivors of childhood cancer: long-term follow-up guidelines and review of the literature. Pediatrics.

[7] Abdel Gader AG (2018). The effect of exercise and nutrition on bone health. J Musculoskelet Surg Res.

[8] Sözen T, Özışık L, Başaran NÇ (2017). An overview and management of osteoporosis. Eur J Rheumatol.

[9] Karimian P, Ebrahimi HK, Jafarnejad S, Delavar MA (2022). Effects of vitamin D on bone density in healthy children: a systematic review. J Family Med Prim Care.

[10] Binu AJ, Cherian KE, Kapoor N, Thomas N, Paul TV (2018). Referral pattern for DXA scanning in a tertiary care centre from southern India. Arch Osteoporos.

[11] Rohani F, Arjmandi Rafsanjani Kh, Bahoush G, Sabzehparvar M, Ahmadi M (2017). Bone mineral density in survivors of childhood acute lymphoblastic leukemia. Asian Pac J Cancer Prev.

[12] Khadilkar AV, Sanwalka NJ, Chiplonkar SA, Khadilkar VV, Mughal MZ (2011). Normative data and percentile curves for dual energy X-ray absorptiometry in healthy Indian girls and boys aged 5-17 years. Bone.

[13] Khera S, Singh J, Kumar A, Kumar R, Kapoor R (2024). Bone mineral density in childhood acute lymphoblastic leukaemia survivors and factors affecting it [in press]. Med J Armed Forces India.

[14] (2023). World Health Organization. BMI-for-age (5-19 years). https://www.who.int/tools/growth-reference-data-for-5to19-years/indicators/bmi-for-age.

[15] Carter DR, Bouxsein ML, Marcus R (1992). New approaches for interpreting projected bone densitometry data. J Bone Miner Res.

[16] Ghassemi A, Banihashem A, Ghaemi N, Elmi S, Erfani Sayyar R, Elmi S, Esmaeili H (2016). Evaluation of bone mineral density in children with acute lymphoblastic leukemia (ALL) and non-Hodgkin's lymphoma (NHL): chemotherapy with/without radiotherapy. Int J Hematol Oncol Stem Cell Res.

[17] Jin HY, Lee JA (2020). Low bone mineral density in children and adolescents with cancer. Ann Pediatr Endocrinol Metab.

[18] Reisi N, Iravani P, Raeissi P, Kelishadi R (2015). Vitamin D and bone minerals status in the long-term survivors of childhood acute lymphoblastic leukemia. Int J Prev Med.

[19] Guru FR, Muzamil J, Bashir S, Mahajan A (2018). Acute lymphoblastic leukemia, the Indian scenario. MOJ Cell Sci Rep.

[20] Mukhopadhyay Sr D, Gupta P, Mukhopadhyay S (2007). Result of childhood acute lymphoblastic leukemia protocol (INCTR) from a developing country. J Clin Oncol.

[21] Greaves MF, Alexander FE (1993). An infectious etiology for common acute lymphoblastic leukemia in childhood?. Leukemia.

[22] Liu YF, Wang BY, Zhang WN (2016). Genomic profiling of adult and pediatric B-cell acute lymphoblastic leukemia. EBioMedicine.

[23] Kong SG, Seo JH, Jun SE, Lee BK, Lim YT (2014). Childhood acute lymphoblastic leukemia with hyperleukocytosis at presentation. Blood Res.

[24] Gustaitė S, Everatt V, Kairienė I, Vaišnorė R, Rascon J, Vaitkevičienė GE (2023). Changes in nutritional status during induction phase and their association with fever and minimal residual disease in paediatric acute lymphoblastic leukaemia. Medicina (Kaunas).

[25] Oliveira LC, Romano LG, Prado-Junior BP, Covas DT, Rego EM, De Santis GC (2010). Outcome of acute myeloid leukemia patients with hyperleukocytosis in Brazil. Med Oncol.

[26] Schündeln MM, Hauffa PK, Munteanu M (2020). Prevalence of osteopathologies in children and adolescents after diagnosis of acute lymphoblastic leukemia. Front Pediatr.

[27] Choudhary A, Chou J, Heller G, Sklar C (2013). Prevalence of vitamin D insufficiency in survivors of childhood cancer. Pediatr Blood Cancer.

[28] Bhattacharya S, Verma N, Kumar A (2020). Prevalence of vitamin D deficiency in childhood acute lymphoblastic leukemia and its association with adverse outcomes during induction phase of treatment. Nutr Cancer.

[29] Tylavsky FA, Smith K, Surprise H (2010). Nutritional intake of long-term survivors of childhood acute lymphoblastic leukemia: evidence for bone health interventional opportunities. Pediatr Blood Cancer.

[30] Tillmann V, Darlington AS, Eiser C, Bishop NJ, Davies HA (2002). Male sex and low physical activity are associated with reduced spine bone mineral density in survivors of childhood acute lymphoblastic leukemia. J Bone Miner Res.

[31] Brignardello E, Felicetti F, Castiglione A (2013). Endocrine health conditions in adult survivors of childhood cancer: the need for specialized adult-focused follow-up clinics. Eur J Endocrinol.

[32] Latoch E, Konstantynowicz J, Krawczuk-Rybak M, Panasiuk A, Muszyńska-Rosłan K (2021). A long-term trajectory of bone mineral density in childhood cancer survivors after discontinuation of treatment: retrospective cohort study. Arch Osteoporos.

[33] van Atteveld JE, Pluijm SM, Ness KK (2019). Prediction of low and very low bone mineral density among adult survivors of childhood cancer. J Clin Oncol.

[34] Toret E, Dural B, Kar YD, Ozdemir ZC, Sivrikoz IA, Bor O (2021). Evaluating bone mineral density in pediatric acute lymphoblastic leukemia survivors: a tertiary care hospital experience. Int J Blood Res Disord.

[35] Halton J, Gaboury I, Grant R (2009). Advanced vertebral fracture among newly diagnosed children with acute lymphoblastic leukemia: results of the Canadian Steroid-Associated Osteoporosis in the Pediatric Population (STOPP) research program. J Bone Miner Res.

[36] Teachey DT, Hunger SP, Loh ML (2021). Optimizing therapy in the modern age: differences in length of maintenance therapy in acute lymphoblastic leukemia. Blood.

[37] Khalid S, Moiz B, Adil SN, Khurshid M (2010). Retrospective review of pediatric patients with acute lymphoblastic leukemia: a single center experience. Indian J Pathol Microbiol.

[38] Choi YJ, Park SY, Cho WK (2013). Factors related to decreased bone mineral density in childhood cancer survivors. J Korean Med Sci.

